# Evaluating trastuzumab deruxtecan in patients with gastrooesophageal adenocarcinoma who are ctDNA and HER2 positive: DECIPHER

**DOI:** 10.1016/j.esmogo.2024.100114

**Published:** 2024-11-28

**Authors:** E. Smyth, D. Griffiths, K. Cozens, S. Ewings, R. Waugh, R.C. Turkington, K. Foley, R. Roy, S. Ngan, R. Owen, D. Chuter, C. Steele, G. Griffiths

**Affiliations:** 1 Oxford University Hospitals NHS Foundation Trust, Churchill Hospital, Oxford, UK; 2 Southampton Clinical Trials Unit, University of Southampton, Centre for Cancer Immunology, University Hospital Southampton, Southampton, UK; 3 Patrick G. Johnston Centre for Cancer Research, Queens University Belfast, Belfast, UK; 4 School of Medicine, Cardiff University, Cardiff, UK; 5 Department of Clinical Oncology, Hull and East Yorkshire Hospitals NHS Trust, Castle Hill Hospital, Hull, UK; 6 Guy’s and St Thomas’ NHS Foundation Trust, Guy’s Hospital, London, UK; 7 Ludwig Institute for Cancer Research, Oxford Nuffield Department of Clinical Medicine, Oxford, UK

**Keywords:** gastrooesophageal adenocarcinoma, oesophageal cancer, gastric cancer, HER2, ctDNA, minimal residual disease

## Abstract

Operable gastrooesophageal adenocarcinoma (GOA) is treated with multimodality therapy which is curative in <50% of patients. Patients in the UK with operable GOA are treated with chemotherapy before and following surgery. Patients who have circulating tumour DNA (ctDNA) present after surgery have worse survival than ctDNA-negative patients. Trastuzumab deruxtecan (T-DXd), a novel human epidermal growth factor receptor 2 (HER2)-targeting antibody–drug conjugate is an effective drug in multiple tumour types and has been licensed to treat advanced HER2-positive GOA that has progressed after chemotherapy and trastuzumab and is European Society for Medical Oncology (ESMO) Guideline recommended. Evaluation of T-DXd in operable but micrometastatic GOA is an attractive option. DECIPHER is a multicentre, phase II trial testing the efficacy of T-DXd in reducing micrometastatic disease burden in HER2-positive GOA patients who are ctDNA positive after neoadjuvant chemotherapy and surgery. Patients will have their resection specimen and plasma analysed to confirm HER2 and ctDNA status post-operatively. Twenty-five ctDNA- and HER2-positive patients will be treated with 6.4 mg/kg T-DXd intravenously every 21 days for a maximum of eight cycles. Study follow-up visits will take place for a maximum of 2 years after treatment, with survival follow-up until the end of the study.

## Highlights

• This was the first study to evaluate T-DXd in ctDNA-positive GOA following surgical resection.
• The trial will indicate whether T-DXd can reduce ctDNA.
• Positive results could inform larger, practice-changing trials.

## Graphical abstract

**Figure:**
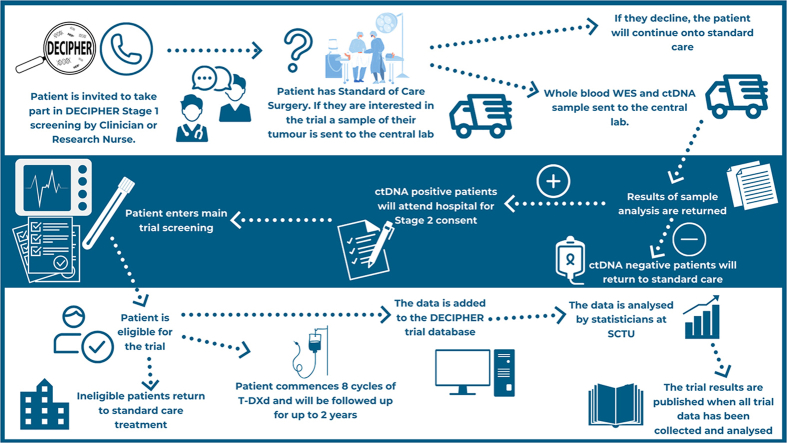

**Table:** Specifications table

Subject area	Medicine and Dentistry
**More specific subject area**	Gastrooesophageal adenocarcinoma
**Name of your trial in progress**	DECIPHER—a single-arm phase II trial of trastuzumab deruxtecan in patients with gastrooesophageal adenocarcinoma cancer who are ctDNA and HER2 positive
**Reagents/tools**	Trastuzumab deruxtecan (T-DXd)
**Trial design**	DECIPHER is a multicentre, phase II trial testing the effect of trastuzumab deruxtecan on reducing micrometastatic disease burden in HER2-positive gastrooesophageal patients who are ctDNA positive after chemotherapy and surgery. To be eligible, participants must have been treated with neoadjuvant chemotherapy before surgery for at least 6 weeks and must be recovered from surgery with no evidence of metastatic disease on post-surgical imaging. Twenty-five patients will be treated with T-DXd at a dose of 6.4 mg/kg intravenously every 21 days for a maximum of eight cycles. The primary endpoint is the percentage of patients who clear ctDNA after 3 months of T-DXd therapy.
**Trial registration**	EudraCT number: 2022-003445-34 ISRCT: ISRCTN15960722 Clinicaltrials.gov: NCT05965479
**Ethics**	There will be a two-stage consent process for DECIPHER. Stage 1 will ask patients to consent to their HER2 and ctDNA status being checked. Before consent is obtained, the patient will be given a full explanation of this stage of the trial and offered a patient information sheet (PIS). Patients can consent to stage 1 by phone (sending the stage 1 informed consent form by post to the research office) or in person. If ctDNA and HER2 positivity is confirmed, the patient will be asked to consent to the main trial. Consent to enter the main trial will be sought from each participant only after a full explanation has been given, a PIS offered, and time allowed for consideration. Signed participant consent will be obtained, using the trial’s main informed consent form, within a hospital setting. The process will be documented in the patient’s medical records. Only site staff named on the delegation log and authorised to do so may obtain consent.
**Value of the trial in progress**	• DECIPHER is the first study to evaluate T-DXd in ctDNA-positive gastrooesophageal adenocarcinoma following surgical resection. • The trial will confirm whether T-DXd can reduce ctDNA in gastrooesophageal adenocarcinoma patients after surgery. • In future, if the results of DECIPHER are positive, these could inform larger, practice-changing trials.

## Description of protocol

### Background

Most patients in the UK with operable gastrooesophageal adenocarcinoma (GOA) are treated with FLOT chemotherapy (comprised of docetaxel, oxaliplatin, and 5-fluoropyrimidine) before and following surgery.^1^ Patients who test circulating tumour DNA (ctDNA) positive after surgery have worse survival than ctDNA-negative patients.^2^ Therefore, the use of novel treatment approaches with an aim to cure this micrometastatic disease is considered to be rational and appropriate. Trastuzumab deruxtecan (T-DXd), a novel human epidermal growth factor receptor 2 (HER2)-targeting antibody–drug conjugate is an effective drug in multiple tumour types and has been licensed in other countries to treat advanced GOA which is HER2 positive and which has progressed after chemotherapy and trastuzumab.^3^

### Methods/design

The DECIPHER trial is a phase II trial evaluating whether T-DXd can reduce micrometastatic disease burden in patients with gastrooesophageal adenocarcinoma who are ctDNA and HER2 positive.

## Objectives

The primary objective is to assess the efficacy of T-DXd in reducing micrometastatic disease burden in HER2-positive GOA patients who are ctDNA positive after chemotherapy and surgery.

Secondary objectives include (i) assessing the impact of T-DXd on ctDNA, (ii) the efficacy of T-DXd in prolonging time to macroscopic disease recurrence, (iii) the efficacy of T-DXd in prolonging survival, and (iv) the safety and tolerability of T-DXd. The quality of life of trial participants will also be assessed using European Organisation for Research and Treatment of Cancer (EORTC)-QLQ-C30, EORTC-QLQ-OG25, and EQ-5D-5L questionnaires.

Tertiary objectives include assessing the effect of T-DXd on ctDNA dynamics and assessing the translational outcomes associated with sensitivity and resistance to T-DXd.

## Study design

DECIPHER is a phase II, open label, multicentre trial of patients with non-metastatic and operable HER2-positive GOA who are ctDNA positive following neoadjuvant chemotherapy and surgery. HER2 positivity will be defined as immunohistochemistry (IHC)3+ or IHC2+/FISH positive on diagnostic biopsy or resection specimen. ctDNA results will be determined using a clinically validated, personalised, tumour-informed assay (CE marked SignateraTM, Natera, Inc., San Carlos, CA).^4^

Similar studies in this field include NCT05034887, which is assessing neoadjuvant T-DXd in a Japanese cohort, and NCT06253650, which will evaluate T-DXd versus T-DXd-5-fluorouracil in ctDNA selected patients post-operatively.

### Treatment

All patients will be treated with T-DXd at a dose of 6.4 mg/kg intravenously every 21 days for a maximum of eight cycles or until disease recurrence. T-DXd is an antibody–drug conjugate composed of a humanized immunoglobulin G1 monoclonal antibody specifically targeting HER2 (trastuzumab), a tetrapeptide-based cleavable linker, and a potent topoisomerase I inhibitor payload (DXd, MAAA-1181a). The schedule of events (Table 1) details the trial treatment schedule and required procedures at each cycle.

**Table 1:** Schedule of observations and procedures

Visit	HER2 and ctDNA confirmation	Screening (within 28 days of the first dose)	Day 1 of cycle 1^a^	Day 8 of cycle 1	Day 1 of cycles 2-8^a^ (infusion ± 2 days)	End of treatment days after day 21 of last cycle ± 2 days)^b^	Follow-up every 12 weeks (±7 days) to 2 years from day 21 of last cycle	Recurrence
Informed consent stage 1	X							
HER2 status	X							
Informed consent stage 2		X						
Genomic DNA (whole blood WES) sample (Natera)	X^c^							
ctDNA sample (Natera)	X^d^				X^e^^,^^f^	X^g^	X^e^	
Translational blood samples (University of Oxford)			X^f^		X^h^^,^^f^	X	X^i^	
Resection tissue sample	X^j^							
Medical history (including smoking and treatment history)		X						
Physical exam (height^k^ and weight), vital signs (temperature, blood pressure, heart rate, respiratory rate, oxygen saturation)		X	X^f^	X	X^f^	X	X	X
ECOG performance status		X	X^f^	X	X^f^		X	X
CT scan (including HRCT scan chest and contrast-enhanced CT scan of abdomen, chest, pelvis)^l^		X			X^m^		X^m^	X^n^
Serum chemistry^o^		X	X^f^	X	X^f^		X	X
Full blood count^o^		X	X^f^	X	X^f^		X	X
Troponin (high sensitivity troponin T)^p^		X				X		
Coagulation tests (INR and aPTT)		X						
Serology (Hep B and Hep C)		X						
Pregnancy for WOCBP (urine)^q^		X	X^f^		X^f^		X^r^	
Urinalysis		X						
Pulmonary function test^s^		X						
ECG		X^s^			X^t^			
ECHO or MUGA		X			X^u^			
Ophthalmology (pupil dilation)^v^		X				X		
Adverse events		X	X	X	X	X	X^w^	X^x^
Concomitant medication^y^		X	X	X	X	X	X	X^x^
Infusion with trastuzumab deruxtecan			X		X			
Recommended tumour biopsy								X
Details of further treatment								X
Survival status								X^z^
Quality-of-life questionnaires (EORTC QLQ-C30, EQ-5D-5L and QLQ-OG25)		X	X^f^		X^f^		X	X
NHS digital form			X^aa^					

aPTT, activated partial thromboplastin time; CT, computed tomography; ctDNA, circulating tumour DNA; DLC, diffusing capacity; DLCO, diffusing capacity of the lung; ECG, electrocardiogram; ECOG, Eastern Cooperative Oncology Group; EORTC, European Organisation for Research and Treatment of Cancer; EOT, end of trial; FEV6, forced expiratory volume in 6 seconds; Hep, hepatitis; HER2, human epidermal growth factor receptor 2; HRCT, high resolution computerised tomography; IMP, investigational medicinal product; INR, international normalised ratio; i.v., intravenous; MUGA, multigated acquisition; NHS; National Health Service; PEF, peak expiratory flow; PFT, pulmonary function test; PI, principal investigator; QLQ, quality of life questionnaire; SCTU, Southampton Clinical Trials Unit; TLC, total lung capacity; WES, whole exome sequencing; WOCBP, woman of childbearing potential.

a Treatment for a maximum of 6 months or until recurrence, cycle = 21 days of i.v. IMP.

b End of treatment visit on day 21 after the last i.v. IMP cycle.

c Genomic DNA samples should be collected before surgery, provided the patients’ HER2 status has been confirmed positive. If unable to collect genomic DNA before surgery, it should be collected as soon as possible after surgery, but no later than the collection of the ctDNA sample. If the genomic DNA sample is not collected before surgery, this will result in a delayed ctDNA result.

d ctDNA blood sample for eligibility to be taken at 4 weeks after surgery and following discharge from hospital and shipped to Natera immediately.

e ctDNA to send to Natera—at day 1 cycle 5 (pre-treatment) and then every 12 weeks for year 1 follow-up and every 6 months in year 2.

f Day 1 examinations and procedures to be completed within 72 h before start of treatment.

g ctDNA taken at EOT only if patient does ‘not’ complete four cycles of T-DXd.

h Samples to be collected on day 1 of cycles 1-5, see laboratory manual for further details.

i Only to be collected at 6 and 12 months’ follow-up visits (follow-up visit 2 and 4).

j Resection sample to be sent to Natera as soon as it is available.

k Height measurement only required at screening.

l Patients must not have an allergy to contrast for CT scans.

m CT scan at screening, then every 12 weeks from C1D1 up to year 2 (±3 days during treatment, ±7 days during follow-up).

n Confirmatory CT to be done within 4-6 weeks after suspected recurrence CT (unless PI determines initial scan is a definitive recurrent cancer).

o Laboratory analysis required at each visit are detailed in the protocol.

p Troponin should also be done if a patient reports signs or symptoms suggesting congestive heart failure, myocardial infarction, or other causes of myocyte necrosis.

q Serum pregnancy test required for eligibility testing at screening, thereafter urine testing can be used before each cycle of treatment.

r At the first and second follow up visit only.

s PFT as a minimum should include spirometry [minimum requirement of: FVC (L), FEV1 % predicted, FEV1/FVC %; optional components include: PEF, FEV6, TLC, DLC]. DLCO will be carried out if feasible, but for patients with prior severe and/or clinically significant pulmonary disorders, DLCO I is strongly encouraged.

t Within 7 days of treatment start date, then repeated at cycle 4 and 8 only.

u Echo at cycle 5 day 1 only.

v Ophthalmologic assessments including visual acuity testing, slit lamp examination, and fundoscopy will be carried out at screening and EOT and as clinically indicated.

w At first follow-up only.

x If recurrence <100 days after last infusion.

y To include those taken 30 days before treatment start and 100 days after trial treatment.

z To be completed every 12 weeks following end of 2 year follow-up or recurrence. A phone call, notes, or primary care contact is acceptable if the patient is not seen in clinic.

aa Form to be sent to SCTU only ‘once’, on cycle 1 day 1.

If required, patients may dose-reduce to level −1 or level −2 as described next. No dose re-escalation is permitted.

- Dose level 0 is 6.4 mg/kg intravenously every 21 days
- Dose level −1 is 5.4 mg/kg intravenously every 21 days
- Dose level −2 is 4.4 mg/kg intravenously every 21 days

T-DXd will be administered using an intravenous (i.v.) bag containing 5% (w/v) dextrose injection infusion solution and delivered through an i.v. administration set with a 0.2 or 0.22 μm filter. The standard infusion time for T-DXd is ∼90 min ± 10 min for the first infusion. If the first infusion is well tolerated and the participant does not experience an infusion-related reaction, then the minimum infusion time for subsequent cycles is 30 min. However, if there are interruptions during the infusion, the total infusion time must not exceed 3 h at room temperature.

T-DXd is classified as a moderately emetogenic anticancer agent, including delayed emesis. Before each dose of T-DXd, participants should be premedicated for prevention of chemotherapy-induced nausea and vomiting. Premedicate with a two- or three-drug combination regimen (e.g. dexamethasone with either a 5-HT3 receptor antagonist or an NK1 receptor antagonist, as well as other drugs as indicated).

There are a set of criteria that should be assessed when considering delaying, discontinuing, or restarting T-DXd treatment in relation to adverse events and infusion-related events (Supplementary Material, available at https://doi.org/10.1016/j.esmogo.2024.100114, details these criteria).

### Setting

DECIPHER will be run in ∼15 National Health Service (NHS) secondary-care hospitals in the UK with the aim of recruiting up to a total of 25 assessable patients. DECIPHER opened to recruitment in April 2024, with a recruitment period of 18 months.

### Sample size and recruitment

Sample size is based on A’Hern’s design^5^^,^^6^ using a binary measure of ctDNA clearance (negativity) as the primary outcome. The level at which the proportion of people achieving ctDNA negativity are felt to be insufficient (*p*_0_) is 0.05 (hence the treatment would not be considered sufficiently active), and the target ctDNA negativity rate (*p*_1_), which would constitute sufficient activity to warrant further investigation, is 0.25; the study is therefore designed to test the null hypothesis that the proportion of people who are ctDNA negative (p) is ≤0.05 against the alternative p ≥ 0.25. Based on a one-sided alpha of 0.05 and power of 0.9, a sample size of 25 is required (with four ctDNA-negative results required to declare sufficient activity of T-DXd). This sample size ensures that the lower limit of the confidence interval will exclude 0.05 with probability 0.90 should the true rate of negativity be 0.25. We will plan to recruit up to 28 participants, to allow for participants not reaching the end of cycle 4.

For the purposes of maximising information on the treatment, we will aim to recruit to target regardless of whether four ctDNA-negative results are obtained before this.

## Ethical and regulatory aspects

The study received ethical approval from North East—Tyne & Wear South Research Ethics Committee on 18 September 2023 (ref: 23/NE/0104) and has Health Research Authority (IRAS 1006810) and UK Medicines and Health Care Product Regulatory Agency (MHRA) approvals. Southampton Clinical Trials Unit (SCTU), a Cancer Research UK core funded and UK Clinical Research Collaboration registered Clinical Trials Unit (CTU), is coordinating the trial. A list of recruiting sites can be obtained from the SCTU. The University of Southampton is the sponsor for the trial and is responsible for all legal requirements of conducting a non-commercial clinical trial of an investigational product. The drug for this trial is provided by AstraZeneca who are responsible for the Good Manufacturing Practice quality drug and is also providing research funding.

The DECIPHER Trial Management Group (TMG) is responsible for overseeing the progress of the trial, including both the clinical and practical aspects. The Chair of the TMG is the Chief Investigator of the trial. The TMG includes representatives with expertise in oncology, radiology, translational science and medical statistics, as well as being supported by two patient and public involvement contributors and SCTU staff involved in the day-to-day running of the trial. An independent Trial Steering Committee has also been established, and an independent Data Monitoring and Ethics Committee (DMEC) comprising two clinicians and a statistician experienced in this research area (but not directly involved in this trial apart from DMEC membership) has been set up. The aim of the independent DMEC is to safeguard the interests of trial participants, monitor the main outcome measures including safety and efficacy, and monitor the overall conduct of the trial. Charters for these groups are available via decipher@soton.ac.uk.

The SCTU has undertaken a risk assessment for the DECIPHER trial, which includes the requirements for monitoring (both central and site). The SCTU undertakes several internal audits of its own systems and processes annually and has routine audits from its sponsor and routine MHRA inspections every 2-3 years.

## Study participants

The DECIPHER trial is currently recruiting patients with non-metastatic and operable HER2-positive gastrooesophageal adenocarcinoma who are ctDNA positive after neoadjuvant chemotherapy and surgery for which the use of T-DXd is the clinically appropriate treatment in the view of the local principal investigator. Participants must have been treated with neoadjuvant chemotherapy before surgery for at least 6 weeks and must have undergone an R0 resection (see Table 2 for full eligibility criteria). No other investigational medicinal products should be received while on study. Immunosuppressive agents, immunosuppressive doses of systemic corticosteroids, concurrent anti-neoplastic therapy, and radiotherapy are prohibited during the study.

**Table 2:** Eligibility criteria for the DECIPHER trial

**Inclusion criteria**
1. Pathologically documented adenocarcinoma of the stomach, gastrooesophageal junction, or lower oesophagus ypTanyNanyM0 with HER2 overexpression (IHC 3+ or IHC 3+/ISH+) based on local tissue testing results
2. ctDNA positive after surgery as per the Signatera assay
3. Capable of giving informed consent before any mandatory study-specific procedures, sampling, or analyses and which includes compliance with the requirements and restrictions listed in the ICF and in this protocol
4. Male and female participants must be at least 18 years of age at the time of signing the ICF
5. Treated with neoadjuvant chemotherapy before surgery for at least 6 weeks
6. Surgical resection with clear margins (R0)
7. Recovered from surgery in the opinion of the investigator
8. No previous treatment with trastuzumab or other HER2-directed therapy
9. No evidence of metastatic disease on post-surgical CT
10. Has an ECOG performance status of 0 to 1
11. Has LVEF ≥ 50% by either ECHO or MUGA scan within 28 days before treatment
12. Has an adequate organ and bone marrow function within 14 days before treatment allocation as follows: Haematological parameters: • Platelet count ≥100 × 10^9^/l (platelet transfusion is not allowed within 1 week before screening assessment, use of thrombopoietin receptor agonists is not allowed within 2 weeks before screening assessment) • Haemoglobin ≥80 g/l. Participants requiring transfusions or growth factor support to maintain haemoglobin ≥80 g/l are not eligible (red blood cell transfusion is not allowed within 1 week before screening assessment) • Absolute neutrophil count ≥1.5 × 10^9^/l (G-CSF administrations are not allowed within 1 week before screening assessment) Liver and renal function: • ALT/AST ≤1.5 × ULN • Total bilirubin ≤1.5 × ULN or <3 × ULN in the presence of documented Gilbert’s syndrome (unconjugated hyperbilirubinemia) • Serum albumin ≥3.0 g/dl • Creatinine clearance ≥50 ml/min as calculated using the Cockcroft–Gault equation Clotting function: • Adequate clotting function INR or prothrombin time and either partial thromboplastin or aPTT ≤1.5 × ULN
13. Reproduction i. Evidence of postmenopausal status or negative serum pregnancy test for females of childbearing potential who are sexually active with a non-sterilised male partner: (i) For women of childbearing potential, a negative result for serum pregnancy test (test must have a sensitivity of at least 25 mIU/ml) must be available at the screening visit and urine β-HCG pregnancy test before each administration of IMP. (ii) Women of childbearing potential are defined as those who are not surgically sterile (i.e. underwent bilateral salpingectomy, bilateral oophorectomy, or complete hysterectomy) or postmenopausal. - Women aged <50 years will be considered postmenopausal if they have been amenorrhoeic for 12 months or more following cessation of exogenous hormonal treatments and if they have LH and FSH levels in the postmenopausal range for the site. - Women aged ≥50 years will be considered postmenopausal if they have been amenorrhoeic for 12 months or more following cessation of all exogenous hormonal treatments, had radiation-induced menopause with last menses >1 year ago, had chemotherapy-induced menopause with last menses >1 year ago. ii. Female participants of childbearing potential who are sexually active with a non-sterilised male partner must use at least one highly effective method of contraception from the time of screening, and must agree to continue using such precautions for 7 months after the last dose of IMP. Not all methods of contraception are highly effective. iii. Female participants must refrain from breastfeeding and must not donate (or retrieve for their own use) ova, from the time of screening, throughout the study treatment period, and for at least 7 months after the last dose of IMP. iv. Complete heterosexual abstinence for the duration of the study and drug washout period in an acceptable contraceptive method if it is in line with the patient’s usual lifestyle (consideration must be given to the duration of the clinical trial); however, periodic or occasional abstinence, the rhythm method, and the withdrawal method are not acceptable methods of contraception. v. Non-sterilised male participants who are sexually active with a female partner of childbearing potential must use a condom with spermicide from screening to 4 months after the final dose of IMP. vi. It is strongly recommended for the female partners of a male participant to also use at least one highly effective method of contraception throughout this period. In addition, male participants should refrain from fathering a child or freezing or donating sperm from screening throughout the study treatment period, and for at least 4 months after the last dose of IMP. vii. Investigators should advise male participants on the conservation of sperm before starting treatment because of the possibility of irreversible infertility/testicular damage due to IMP administered in this study. viii. Female subjects must not donate, or retrieve for their own use, ova from the time of enrolment and throughout the study treatment period, and for at least 7 months after the final study drug administration. They should refrain from breastfeeding throughout this time. Preservation of ova before enrolment in this study can be discussed with the patient if clinically appropriate to do so.
**Exclusion criteria**
14. Uncontrolled intercurrent illness, including but not limited to, ongoing or active infection, or psychiatric illness/social situations that would limit compliance with study requirement, substantially increase risk of incurring AEs or compromise the ability of the participant to give written informed consent.
15. Participants with a medical history of myocardial infarction within 6 months before treatment or symptomatic CHF (New York Heart Association Class II IV), unstable angina pectoris, clinically important cardiac arrhythmias, or a recent (<6 months) cardiovascular event, including myocardial infarction, unstable angina pectoris, and stroke. Participants with troponin levels above ULN at screening (as defined by the manufacturer), and without any myocardial-related symptoms, should have a cardiologic consultation before enrolment to rule out myocardial infarction.
16. Correct QT interval (QTcF) prolongation to >470 ms (females) or > 450 ms (males) based on average of the screening triplicate 12-lead ECG.
17. History of (non-infectious) ILD/pneumonitis, current ILD/pneumonitis, or, where suspected, ILD pneumonitis cannot be ruled out by imaging at screening.
18. Any of the following: (i) Lung-specific intercurrent clinically significant illnesses including, but not very limited to, any underlying pulmonary disorder (e.g. clinically significant pulmonary emboli within 3 months of treatment, severe asthma, severe chronic obstructive pulmonary emboli within 3 months of treatment, severe asthma, severe chronic obstructive pulmonary disease, restrictive lung disease, clinically significant pleural effusion, etc.) (ii) Any autoimmune, connective tissue, or inflammatory disorders with pulmonary involvement (e.g. rheumatoid arthritis, Sjogren’s, sarcoidosis, etc.), where there is documented, or suspicion of pulmonary involvement at the time of screening. (iii) Prior pneumonectomy (complete).
19. Uncontrolled infection requiring intravenous antibiotics, antivirals, or antifungals.
20. Multiple primary malignancies within the previous 3 years, except adequately resected non-melanoma skin cancer, curatively treated *in situ* disease, or other solid tumours curatively treated.
21. A pleural effusion, ascites, or pericardial effusion that requires drainage and peritoneal shunt.
22. Unresolved toxicities from previous anticancer therapy, defined as toxicities (other than alopecia) not yet resolved to grade ≤1 or baseline. The following exemption will apply; stable chronic G2 toxicity which in the opinion of the investigator is not reasonably expected to be exacerbated by treatment with study drugs.
23. Known allergy or hypersensitivity to T-DXd or any of the study drug excipients.
24. History of severe hypersensitivity reactions to other monoclonal antibodies.
25. Pregnant or breastfeeding female participants, or other participants who are planning to become pregnant.
26. Involvement in the planning and/or the conduct of the study.
27. Has substance abuse or any other medical conditions, that may, in the opinion of the Investigator, interfere with the subject’s participation in the clinical study or evaluation of the clinical study results.
28. Receipt of live, attenuated vaccine within 30 days before the first dose of T-DXd. Note: Patients, if enrolled, should not receive live vaccine during the study and up to 30 days after the last dose of IMP.
29. Active primary immunodeficiency, known HIV infection, or active hepatitis B or C infection. Patients positive for HCV antibody are eligible only if polymerase chain reaction is negative for HCV RNA.
30. Judgement by the Investigator that the participant should not participate in the study, if the participant is unlikely to comply with study procedures, restrictions, and requirements.

β-HCG, beta-human chorionic gonadotropin; AEs, adverse events; ALT, alanine aminotransferase; aPTT, activated partial thromboplastin time; AST, aspartate aminotransferase; CHF, congestive heart failure; CT, computed tomography; ctDNA, circulating tumour DNA; ECG, electrocardiogram; ECOG, Eastern Cooperative Oncology Group; ECHO, echocardiogram; FSH, follicle-stimulating hormone; G-CSF, granulocyte colony-stimulating factor; HCV, hepatitis C virus; HER2, human epidermal growth factor receptor 2; HIV, human immunodeficiency virus; ICF, informed consent form; IHC, immunohistochemistry; ILD, interstitial lung disease; IMP, investigational medicinal product; INR, international normalised ratio; ISH, in situ hybridisation; LH, luteinizing hormone; LVEF, left ventricular ejection fraction; MUGA, multigated acquisition; T-DXd, trastuzumab deruxtecan; ULN, upper limit of normal.

## Study procedure

### Stage 1

There will be a two-stage consent process for participation in the DECIPHER trial. Stage 1 will ask patients to consent to their HER2 status being checked. This may have already been done as part of standard care and will be recorded in the patient notes. If it has not been done, the archival tumour tissue (collected at diagnosis) will be analysed locally to determine HER2 status. HER2 status must be confirmed positive before surgery. If patients are HER2 positive, they will be asked to consent to a resection tumour sample, obtained during standard-of-care surgery, and blood samples taken at a minimum of 4 weeks after surgery, provided the patient has been discharged to be sent to Natera for ctDNA analysis. The ctDNA sample can be taken up to 8 weeks post-surgery, where required. Should the analysis show ctDNA positivity, the patient will be asked if they wish to participate in the main trial. Before Stage 1 consent is obtained, the patient will be given a full explanation of this stage of the trial and offered a patient information sheet (PIS). Patients can consent to Stage 1 by phone (sending the Stage 1 informed consent form by post to the research office) or in person. To be eligible to consent to Stage 1, patients must have the following:

(i) Pathologically documented adenocarcinoma of the stomach, gastrooesophageal junction, or oesophagus TanyNanyM0
(ii) Archival tumour tissue available to be analysed locally for HER2 or prior results of HER2 testing being available.

### Stage 2

If ctDNA positivity is confirmed, the patient will be asked to consent to Stage 2. Stage 2 consent to enter the main trial will be sought from each participant only after a full explanation has been given, a PIS offered, and time allowed for consideration. Signed participant consent will be obtained, using the Stage 2 informed consent form, within a hospital setting. The process will be documented in the patient’s medical records. Only site staff named on the delegation log and authorised to do so may obtain consent. Patients may refuse to participate without giving reasons and this will not prejudice their future treatment.

The trial’s informed consent form details the consent provisions for collection and use of participant data and biological specimens in future research. Following consent, patients will enter Stage 2 screening and, if eligible, will continue on to treatment and follow-up, following the schedule of events (Table 1). T-DXd should be started up to 12 weeks post-operatively to facilitate recovery from surgery.

Participants are free to withdraw consent from the study at any time without providing a reason, and without their medical care or legal rights being adversely affected.

### Translational research

Tumour and plasma samples will be examined to assess the effect of T-DXd on ctDNA dynamics and to assess translational outcomes associated with sensitivity and resistance to T-DXd.

## Statistical analysis

### Primary endpoint

The primary endpoint of proportion (*p*) of ctDNA negativity after four cycles of T-DXd will be presented with corresponding exact one-sided (lower bound) and two-sided 95% confidence intervals and *P* value from a one-sided test against the null hypothesis of *P*_0_ = 0.05. Using the ICH-E9 addendum estimands framework,^5^^,^^6^ the targeted estimand is the effect of T-DXd on ctDNA clearance in those participants who complete four cycles of treatment, who would not withdraw from the trial due to treatment toxicity or other reasons or die of non-cancer-related reasons. This means that anyone withdrawing/withdrawn from treatment before providing primary endpoint data will be discounted from the final analysis (principal stratum strategy). In the case of death before primary endpoint data being collected, a cancer recurrence-related death will count as a failure (i.e. ctDNA positive; composite endpoint strategy), and other deaths will be excluded from the analysis (principal stratum strategy). Those who do not initiate treatment will also be excluded (principal stratum strategy). Participants who experience treatment delays and/or missed doses and/or dose reduction will be included as normal in the analysis (treatment policy strategy).

Two prospective studies PLAGAST and PANDA demonstrate that ctDNA is a strong predictor of early recurrence and response to treatment.^7^^,^^8^ ctDNA can dynamically reflect tumour burden, predict treatment efficacy, and correlate with long-term outcomes like recurrence and survival. It is notable that DECIPHER is the first study evaluating ctDNA as an endpoint in the adjuvant setting, and the trial is a phase II trial which can be considered exploratory.

The decision to recruit in the adjuvant setting was driven by the fact that treatment stratification preoperatively had less evidence for ctDNA-guided therapy and the fact that patients in the adjuvant setting who are ctDNA positive can be considered to have tumours which have ‘failed’ neoadjuvant FLOT and are therefore in need of a different treatment.

### Secondary endpoint

The proportion of patients with ctDNA negativity after each cycle (for cycles 1-5), plus each time point post-treatment for the first year and month 18 and 24 in the second year of follow-up, will be presented, alongside one-sided exact confidence intervals (as per A’Hern’s design). Disease-free survival and overall survival (OS) will be presented as medians, and percentages disease free and alive, respectively, at 12 and 24 months (plus 18 months for OS), derived from the Kaplan–Meier estimate. Estimates will be presented alongside corresponding 95% confidence intervals. Participants who do not experience disease recurrence or death will be censored at the last time point in which data is available for these endpoints (including those who withdraw, for whom the assumption will be non-informative censoring; reasons for withdrawal will, however, be presented to judge the suitability of this assumption). Symptoms and quality-of-life data will be presented descriptively by time point.

Adverse event data will be presented for everyone enrolled in the trial, using frequency of events and percentage of participants affected. Adverse events will be presented in summary using the Common Terminology Criteria for Adverse Events v5.0 grade, and by standard of care and preferred terms.

There are no plans for any formal interim analysis to be conducted during the DECIPHER trial. The DMEC will monitor safety data throughout the trial.

## End of the trial

The end of trial is defined as the time when all expected data visits have taken place and the database has been locked following the resolution of data queries, as far as possible, as agreed by the TMG.

The anticipated date of study completion is June 2028.
