# Supplementary material for: Evaluating trastuzumab deruxtecan in patients with gastrooesophageal adenocarcinoma who are ctDNA and HER2 positive: DECIPHER
Source: ESMO Gastrointest Oncol. 2024 Nov 28;6:100114. doi: 10.1016/j.esmogo.2024.100114 (PMC12836495; doi:10.1016/j.esmogo.2024.100114)
Supplement: Supplementary Material [file mmc1.docx]

**Additional File 1 - Trastuzumab Deruxtecan Dose Delays and Modifications**

| **Infusion reaction** | |
| --- | --- |
| Grade 1 (Mild transient reaction;  infusion interruption not indicated;  intervention not indicated) | If infusion related reaction (such as fever and chills, with and without  nausea/vomiting, pain, headache, dizziness, dyspnea, and/or  hypotension) is observed during administration, the infusion rate should  be reduced by 50%, and participants should be closely monitored.  If no other reactions appear, the subsequent infusion rate could be  resumed at the initial planned rate. |
| Grade 2 (Therapy or infusion  interruption indicated but responds  promptly to symptomatic treatment  (eg, antihistamines, nonsteroidal  anti-inflammatory drugs (NSAIDs),  narcotics, and/or IV fluids);  prophylactic medications indicated  for ≤ 24 hrs) | Administration of T-DXd should be interrupted and symptomatic  treatment started (eg, antihistamines, NSAIDs, narcotics, and/or IV  fluids).  If the event resolves or improves to Grade 1, infusion can be restarted  at a 50% reduced infusion rate.  Subsequent administrations should be conducted at the reduced rate. |
| Grade 3 or 4 (Prolonged or  life-threatening consequences;  urgent intervention indicated) | Administration of T-DXd should be discontinued immediately and  permanently.  Urgent intervention indicated. Antihistamines, steroids, epinephrine,  bronchodilators, vasopressors, intravenous fluid therapy, oxygen  inhalation, etc, should be administered. |
| **Haematological toxicity** | |
| Hematologic toxicity (if supportive therapy fails [as clinically indicated and according to local practice],  consider additional toxicity management as below). For any Grade 4 hematological toxicity with significant clinical symptoms that does not resolve with treatment within 4 weeks, resuming IMP may be possible if the toxicity resolves, in consultation with the Chief Investigator. | |
| **Neutrophil count decreased** | |
| Grade 3 | Delay dose until resolved to ≤ Grade 2, then maintain dose |
| Grade 4 | Delay dose until resolved to ≤ Grade 2, then reduce dose 1 level and remain at this level for subsequent cycles. No re-escalation is permitted. |
| Febrile neutropenia (absolute neutrophil count < 1 × 109/L, fever > 38.3°C, or a sustained temperature of ≥ 38 °C for more than 1 hour) | Delay dose until resolved, then reduce dose by 1 level |
| Lymphocyte count decreased | |
| Grade 1 to Grade 3 lymphopenia | No dose modification |
| Grade 4 (< 0.2 × 109/L) | Delay dose until resolved to ≤ Grade 2:  If resolved in ≤ 14 days from day of onset, then maintain dose  If resolved in > 14 days from day of onset, then reduce dose 1 level |
| **Anaemia** | |
| Grade 3 (Hemoglobin < 8.0 g/dL);  transfusion indicated | Delay dose until resolved to ≤ Grade 2, then maintain dose |
| Grade 4 Life threatening  consequences; urgent intervention  indicated | Delay dose until resolved to ≤ Grade 2, then reduce dose 1 level |
| **Platelet count decreased** | |
| Grade 3 (platelets < 50 to 25 ×  109/L) | Delay dose until resolved to ≤ Grade 1:  If resolved in ≤ 7 days from day of onset, then maintain dose  If resolved in > 7 days from day of onset, then reduce dose 1 level |
| Grade 4 (platelets < 25 × 109/L) | Delay dose until resolved to ≤ Grade 1, then reduce dose 1 level |
| **Cardiac toxicity** | |
| Symptomatic CHF | Discontinue participant from study treatment |
| Grade 2: Decrease in LVEF 10% to  20% (absolute value), but LVEF  > 45% | Continue treatment with T-DXd |
| Grade 2: LVEF 40% to ≤ 45% and  decrease is < 10% (absolute value)  from baseline | Continue treatment with T-DXd  Repeat LVEF assessment within 3 weeks |
| Grade 2: LVEF 40% to ≤ 45% and  decrease is 10% to 20% (absolute  value) from baseline | Interrupt T-DXd dosing  Repeat LVEF assessment within 3 weeks  If LVEF has not recovered to within 10% (absolute value) from  baseline, discontinue participant from study treatment  If LVEF recovers to within 10% from baseline, resume IMP treatment |
| Grade 3: LVEF < 40% or > 20%  (absolute value) drop from baseline | Interrupt T-DXd dosing  Repeat LVEF assessment within 3 weeks  If LVEF < 40% or > 20% drop from baseline is confirmed, discontinue  participant from study treatment  If LVEF has recovered to > 40% and decrease is < 20% from baseline,  follow appropriate guidelines above |
| Electrocardiogram QTC prolonged | |
| Grade 3 (Average QTc > 500 ms or  > 60 ms change from baseline) | Delay dose until resolved to ≤ Grade 1 (corrected QT ≤ 480 ms),  determine if another medication the participant was taking may be  responsible and can be adjusted or if there are any changes in serum  electrolytes that can be corrected, then if attributed to T-DXd, reduce  dose 1 level |
| Grade 4 (Torsade de pointes or  polymorphic ventricular  tachycardia or signs/symptoms of  serious arrhythmia) | Discontinue participant from study treatment |
| **Pulmonary toxicity** | |
|  | Work-up of suspected ILD/pneumonitis:  If a participant develops radiographic changes potentially consistent  with ILD/pneumonitis or develops an acute onset of new or worsening  pulmonary or other related signs/symptoms such as dyspnea, cough or  fever, rule out ILD/pneumonitis  Evaluations should include:  • High resolution CT  • Pulmonologist consultation (infectious disease consultation as  clinically indicated)  • Blood culture and complete blood count. Other blood tests could  be considered as needed  • Consider bronchoscopy and bronchoalveolar lavage if clinically  indicated and feasible  • Pulmonary function tests and pulse oximetry (SpO2)  • Arterial blood gases if clinically indicated  • One blood sample collection for PK and exploratory biomarker  analysis as soon as ILD is suspected, if feasible  Other tests could be considered, as needed.  If the AE is confirmed to have an aetiology other than treatment-related  ILD/pneumonitis, follow the appropriate management guidance  If another aetiology for the AE cannot be identified and it could be related to T-DXd, then follow the ILD/pneumonitis management  guidance as outlined below.  All events of ILD regardless of severity or seriousness will be followed  until resolution. |
| Grade 1 Management: | Monitor and closely follow-up in 2 to 7 days for onset of clinical  symptoms and pulse oximetry  Consider follow-up imaging in 1-2 weeks (or as clinically indicated)  Consider starting systemic steroids (eg, at least 0.5 mg/kg/day  prednisone or equivalent) until improvement, followed by gradual taper  over at least 4 weeks  If worsening of diagnostic observations despite initiation of  corticosteroids, then follow Grade 2 guidelines*  Dose modification:  The administration of T-DXd must be interrupted. T-DXd can be  restarted only if the event is fully resolved to Grade 0:  If resolved in ≤ 28 days from day of onset, then maintain dose  If resolved in > 28 days from day of onset, then reduce dose 1 level  However, if the event Grade 1 ILD occurs beyond cycle Day 22 and  has not resolved within 49 days from the last infusion, the drug should  be discontinued  *If a participant is asymptomatic, then the subject should still be  considered as Grade 1 even if steroid treatment is given |
| Grade 2 | Dose Modification:  Permanently discontinue participant from study treatment.  Management:  Promptly start and treat with systemic steroids (eg, at least 1 mg/kg/day  prednisone or equivalent) for at least 14 days followed by a gradual  taper over at least 4 weeks  Monitor symptoms closely  Re-image as clinically indicated  If worsening or no improvement in clinical or diagnostic observations  in 3-5 days:  Consider increasing dose of steroids (eg, 2 mg/kg/day prednisone or  equivalent) and switching treatment administration to IV (eg,  methylprednisolone)  Re-consider additional work-up for alternative etiologies as described  above  Escalate care as clinically indicated |
| Grade 3 or 4 | Dose modification:  Permanently discontinue participant from study treatment.  Management:  Hospitalization required  Promptly initiate empiric high-dose methylprednisolone IV treatment  (eg, 500-1000 mg/day for 3 days), followed by at least 1.0 mg/kg/day  of prednisone (or equivalent) for at least 14 days, followed by a gradual  taper over at least 4 weeks  Re-image as clinically indicated  If still no improvement within 3-5 days:  Re-consider additional work-up for alternative etiologies as described  above  Consider other immunosuppressants and/or treat per local practice |
| **Ocular** | |
| Grade 3 | Delay dose until resolved to ≤ Grade 1:  If resolved in ≤ 7 days from day of onset, then maintain dose  If resolved in > 7 days from day of onset, then reduce dose 1 level |
| Grade 4 | Discontinue participant from study treatment |
| **Blood creatinine increased** | |
| Grade 3 (> 3.0 to 6.0 × ULN) | Delay dose until resolved to ≤ Grade 2 or baseline, then reduce dose  1 level |
| Grade 4 (> 6.0 × ULN) | Discontinue participant from study treatment |
| **Hepatic toxicity** | |
| **Aspartate aminotransferase (AST) or alanine aminotransferase (ALT) with simultaneous blood**  **bilirubin increased** | |
| Grade 2 (> 1.5 to 3.0 × ULN if  baseline was normal; > 1.5 to  3.0 × baseline if baseline was  abnormal) | If no documented Gilbert’s syndrome or liver metastases at baseline,  delay dose until resolved to ≤ Grade 1:  If resolved in ≤ 7 days from day of onset, then maintain dose  If resolved in > 7 days from day of onset, then reduce dose 1 level  If documented Gilbert’s syndrome or liver metastases at baseline,  continue study treatment |
| Grade 3 (> 3.0 to 10.0 × ULN if  baseline was normal; > 3.0 to  10.0 × baseline if baseline was  abnormal) | If no documented Gilbert’s syndrome or liver metastases at baseline,  repeat testing within 3 days. Delay dose until resolved to ≤ Grade 1:  If resolved in ≤ 7 days from day of onset, then reduce dose 1 level  If resolved in > 7 days from day of onset, then discontinue T-DXd  If documented Gilbert’s syndrome or liver metastases at baseline,  repeat testing within 3 days. Delay dose until resolved to < Grade 2:  If resolved in ≤ 7 days from day of onset, then reduce dose 1 level  If resolved in > 7 days from day of onset, then discontinue T-DXd |
| Grade 4 (> 10.0 × ULN if baseline  was normal; > 10.0 × baseline if  baseline was abnormal) | Discontinue participant from study treatment |
| **Blood alkaline phosphatase increased** | |
| Grade 3 (> 5.0 to 20.0 × ULN if  baseline was normal; > 5.0 to  20.0 × baseline if baseline was  abnormal)  Or Grade 4 (> 20.0 × ULN if  baseline was normal; > 20.0 ×  baseline if baseline was abnormal) | No modification unless determined by the Investigator to be clinically  significant or life-threatening. |
| **Gastrointestinal** | |
| **Nausea** | |
| Grade 3 Delay dose until resolved to ≤Grade 1: | If resolved in ≤ 7 days from day of onset, then maintain dose  If resolved in > 7 days from day of onset, then reduce dose 1 level |
| **Diarrhoea/Colitis** | |
| Grade 3 | Delay dose until resolved to ≤Grade 1:  If resolved in ≤ 3 days from day of onset, then maintain dose  If resolved in > 3 days from day of onset, then reduce dose 1 level |
| Grade 4 | Discontinue participant from study treatment |
| **Stomatitis/Mucosal inflammation** | |
| **Follow the SOC for managing stomatitis/mucosal inflammation**  **Educate subject on importance of good oral hygiene** | |
| Grade 1 | Initiate supportive treatment  Maintain dose |
| Grade 2 | Initiate supportive treatment  Reduce dose if clinically indicated |
| Grade 3 | Delay dose until resolved to ≤Grade 1:  If resolved in ≤ 7 days, maintain dose  If resolved in > 7 days, reduce dose by 1 level |
| Grade 4 | Discontinue participant from study treatment |
| **Other laboratory adverse events** | |
| Grade 3 | Delay dose until resolved to ≤ Grade 1 or baseline level:  If resolved in ≤ 7 days from day of onset, then maintain dose  If resolved in > 7 days from day of onset, then reduce dose 1 level |
| Grade 4 | Discontinue participant from study treatment |
| **Other non-laboratory adverse events** | |
| Grade 3 | Delay dose until resolved to ≤ Grade 1 or baseline:  If resolved in ≤ 7 days from day of onset, then maintain dose  If resolved in > 7 days from day of onset, then reduce dose 1 level |
| Grade 4 | Discontinue participant from study treatment |
